# Metabolomics reveals the metabolic shifts following an intervention with rye bread in postmenopausal women- a randomized control trial

**DOI:** 10.1186/1475-2891-11-88

**Published:** 2012-10-22

**Authors:** Ali A Moazzami, Isabel Bondia-Pons, Kati Hanhineva, Katri Juntunen, Nadja Antl, Kaisa Poutanen, Hannu Mykkänen

**Affiliations:** 1 Department of Chemistry, Swedish University of Agricultural Sciences, P.O. Box 7015, Uppsala, SE, 75007, Sweden; 2 Department of Food Science, Swedish University of Agricultural Sciences, Uppsala, Sweden; 3 Department of Public Health and Clinical Nutrition, Clinical Nutrition, Food and Health Research Centre, University of Eastern Finland, Kuopio Campus, P.O. Box 1627, Kuopio, FIN 70211, Finland; 4 VTT Technical Research Centre of Finland, P.O. Box 1000, Tietotie 2, Espoo, FI-02044, Finland; 5 City of Kuopio, Health Care Services, Health Care Unit, Suokatu 40 B, Kuopio, FI-70110, Finland

**Keywords:** Whole grain rye, Wheat, Metabolomics, NMR, Amino acid, Homocysteine, Postmenopausal, Single carbon metabolism

## Abstract

**Background:**

Epidemiological studies have consistently shown that whole grain (WG) cereals can protect against the development of chronic diseases, but the underlying mechanism is not fully understood. Among WG products, WG rye is considered even more potent because of its unique discrepancy in postprandial insulin and glucose responses known as the rye factor. In this study, an NMR-based metabolomics approach was applied to study the metabolic effects of WG rye as a tool to determine the beneficial effects of WG rye on human health.

**Methods:**

Thirty-three postmenopausal Finnish women with elevated serum total cholesterol (5.0-8.5 mmol/L) and BMI of 20–33 kg/m^2^ consumed a minimum of 20% of their daily energy intake as high fiber WG rye bread (RB) or refined wheat bread (WB) in a randomized, controlled, crossover design with two 8-wk intervention periods separated by an 8-wk washout period. At the end of each intervention period, fasting serum was collected for NMR-based metabolomics and the analysis of cholesterol fractions. Multilevel partial least squares discriminant analysis was used for paired comparisons of multivariate data.

**Results:**

The metabolomics analysis of serum showed lower leucine and isoleucine and higher betaine and N,N-dimethylglycine levels after RB than WB intake. To further investigate the metabolic effects of RB, the serum cholesterol fractions were measured. Total- and LDL-cholesterol levels were higher after RB intake than after WB (p<0.05).

**Conclusions:**

This study revealed favorable shifts in branched amino acid and single carbon metabolism and an unfavorable shift in serum cholesterol levels after RB intake in postmenopausal women, which should be considered for evaluating health beneficial effects of rye products.

## Background

Whole grains (WG) are a rich source of fiber and bioactive compounds, including tocopherols, B vitamins, minerals, phenolic acids, and phytoestrogens [1]. There is growing evidence that WG cereals protect against the development of chronic diseases such as type 2 diabetes (T2D) [2,3], cardiovascular disease (CVD) [4-6], and certain cancers [7-10]. WG rye, which is the traditional WG bread consumed in northern Europe, is believed to potentially exert even more positive effects than other WG cereals due to the so-called ‘rye factor’ [11]. For example, WG rye bread intake was the factor most consistently associated with lower mortality in Danish men in a recent prospective study with 12 years of follow-up [12].

Postmenopausal women display decreased insulin sensitivity, insulin secretion, and hepatic insulin extraction [13]. They therefore potentially have more pronounced susceptibility to diabetes and CVD, which makes them an interesting target population in examining the physiological effects of potentially protective foods against CVD and T2D, such as WG rye. There is growing evidence that T2D and related conditions are characterized by a broad perturbation of metabolic physiology involving considerable changes in lipid and amino acid metabolism in addition to glucose [14]. This new evidence is prompting the application of methods monitoring a broad range of molecular species, i.e. metabolomics, to study the beneficial effects of potentially health-promoting foods.

The application of metabolomics to well-designed controlled intervention studies can be a useful tool to elucidate the complex physiological effects of WG rye, which might help in understanding their beneficial effects on human health. Metabolomics has proven to be successful in mechanistic understanding of beneficial health effects of WG products using both NMR and LC/MS approaches [15-18].

Lipidomics and GC-MS based metabolomics were recently applied to compare the effects of a high-fiber WG rye bread diet and a refined wheat bread diet on the lipid and metabolic profiles of postmenopausal women [16]. However, in view of the differences in the analytical specificity of different platforms, the application of various analytical platforms, i.e. lipidomics, LC-MS-based metabolomics, and NMR, is necessary to expand the number of detectable metabolites and thus achieve a more comprehensive understanding of modulation in metabolic profile following an intervention.

Therefore the present study looked for further changes in the serum metabolic profile of postmenopausal women from the same intervention as reported by our group [16,19] using the application of NMR-based metabolomics.

## Methods

### Human intervention

Forty-three postmenopausal women were recruited for the intervention (18) through an advertisement in a local newspaper. The inclusion criteria were: BMI 20–33 kg/m^2^, serum total cholesterol concentration 5.0-8.5 mmol/L, non-HDL cholesterol concentration 3.5-6.5 mmol/L, and serum triglyceride concentration <2.5mmol/L. The exclusion criteria included the use of lipid-lowering drugs, laxatives, or corticosteroid medication. Women diagnosed with diabetes mellitus were also excluded. Postmenopausal status was confirmed by measuring the concentration of follicle-stimulating hormone concentration in serum (>30 U/L). All participants provided written informed consent for the study and the study protocol was approved by the Ethics Committee of Kuopio University Hospital. Four women discontinued the study for personal or health reasons, so the final number of study subjects was 39. However, serum samples were available for only 33, so the data for metabolomics and cholesterol analysis are reported for n = 33. The order of the test bread intervention periods based on available serum samples was WB-RB for 17 women and RB-WB for 16 women. At the beginning of the study, participants (58.8 ± 5.8 years old) had body mass index (kg/m^2^) 27.2 ± 3.2, systolic blood pressure (mm Hg) 127.5 ± 17.9, and diastolic blood pressure (mm Hg) 79.8 ± 8.3.

The study protocol is reported in detail in a previous paper [19]. The present study was conducted in a randomized crossover design with two 8-wk intervention periods separated by an 8-wk washout period. The first intervention period was preceded by a 2- to 3-wk run-in period and afterwards participants were randomly assigned into either an 8-wk high fiber rye bread (RB) period or an 8-wk refined wheat bread (WB) period. At the beginning of the run-in period, participants were advised to maintain their lifestyle habits, regular medication, and body weight throughout the study. They were also instructed to avoid foods containing plant stanols/sterols, probiotics, or products that affect bowel function. During the washout period, participants consumed their habitual diet. During the intervention periods, a minimum of 20% of daily energy intake was provided by the test bread, while consumption of other cereal products, such as sweet pastries and porridges, was limited to 1 portion/d. A minimum of 4–5 portions of the test bread had to be eaten daily. Each portion of rye bread weighed 24–28 g and each portion of wheat bread 21–25 g. The high-fiber rye bread (RB) (≈ 17% dietary fiber) was prepared by increasing the content of rye bran in the breads, which caused a major increase in the content of insoluble fiber. The nutrient composition of the RB and WB used in the study was analyzed at the Technical Research Centre of Finland (VTT) and has been reported previously [19]. Four-day food records that included one weekend day (consecutive days) were kept during the run-in period and during wk 4–6 in both bread intervention periods. Food records were analyzed by a clinical nutritionist using MicroNutrica dietary analysis software (version 2.0; Finnish Social Insurance Institute).

Serum and plasma samples were collected from 12-h fasting women at the end of the run-in and test bread periods. Blood drawn from an antecubital vein was collected in prechilled vacuum tubes to prepare serum. The samples were stored in 2-mL cryotubes at −80°C until analysis.

### Metabolomics analysis

NMR-based metabolomics analysis of serum samples collected at the end of each intervention period was performed using previously described methods after slight modification [15,20,21]. Nanosep centrifugal filters with 3 kDa cutoff (Pall Life Science, Port Washington, NY) were washed 8 times with 0.5 mL water at 4000 g and 36°C to remove glycerol from the filter membrane. The filters were kept at 4°C and then 400 μL of plasma sample were filtered at 10,000 g and 4°C. Phosphate buffer (150 μL, 0.4 mol/L, pH 7.0), D_2_O (45 μL) and sodium-3-(trimethylsilyl)-2,2,3,3-tetradeuteriopropionate (TSP, 30 μL, 5.8 mmol/L) (Cambridge Isotope Laboratories, Andover, MA) as an internal standard were added to 375 μL plasma filtrate to ensure quantitative measurements of metabolites captured by NMR. The mixture was then used for NMR analysis. All NMR analyses of plasma samples were performed on a Bruker spectrometer operating at 400 MHz (Karlsruhe, Germany). ^1^H NMR spectra of plasma samples were obtained using zgesgp pulse sequence (Bruker Spectrospin Ltd.) at 25°C with 360 scans and 32,768 data points over a spectral width of 4789.27 Hz. Acquisition time was 3.42 s and relaxation delay was 3.0 s. The discriminating NMR signals were identified primarily using the NMR Suite 7.1 library (ChenomX Inc, Edmonton, Canada), Human Metabolome Data Base, Biological Magnetic Resonance Data Bank, and in the event of multiplicity were confirmed with 2D NMR and previously published data [22].

### Biochemical analyses

Complementary biochemical analyses of cholesterol and triglycerides in serum were performed to further investigate the metabolic effects of RB. Serum samples collected at the end of the run-in and test bread periods were used for analysis of blood lipids. Lipoproteins were separated by ultracentrifugation for 18 h at density 1.006 kg/L to remove the VLDL fraction. HDL in the infranatant was separated from LDL by precipitation of LDL. LDL was calculated as the difference between the mass of cholesterol in the infranatant and HDL. Serum total cholesterol and triglycerides were measured by an enzymatic colorimetric method with commercial kits (Monotest Cholesterol and Triglyceride GPO-PAP, Boehringen Mannheim GmbH Diagnostica) using an automated instrument (Kone Specific Clinical Analyzer, Kone Ltd, Espoo, Finland). The within-assay CV for triglycerides using 4 standards was 1.7-2.4% and the between-assay CV was 1.3-1.6% (3 standards). The corresponding values for HDL cholesterol were 1.4% (1 standard) and 6.3% (1 standard).

### Statistical analysis

The NMR spectral data were processed using Bruker Topspin 1.3 software and were Fourier-transformed after multiplication by a line broadening of 0.3 Hz and referenced to TSP at 0.0 ppm. Spectral phase and baseline were corrected manually. Each spectrum was integrated using Amix 3.7.3 (Bruker BioSpin GmbH, Rheinstetten) into 0.01 ppm integral regions (buckets) between 0.75-8.5 ppm, in which areas between 4.25-6.75 ppm containing urea and residual water were removed. Each spectral region was then normalized to the intensity of internal standard (TSP).

**Figure 1:**
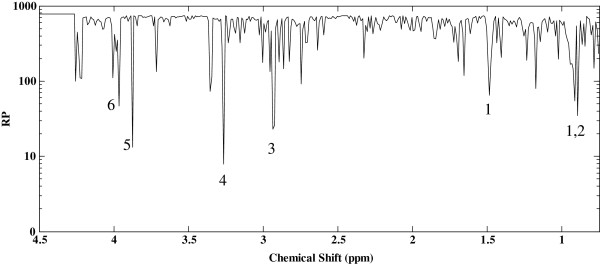
**Rank product (RP**^**1/20**^**) from multilevel partial least-squares discriminant analysis models of the comparsion between the NMR spectra of serum after the intake of refined wheat bread vs the intake of whole grain rye bread in postmeopuasal women (n=33).** 1: Isoleusine; 2: Leusine; 3: N,N-dimethylglycine; 4: Betaine; 5: an overlapping signal with glucose at 3.875 ppm; 6: small signal in an overlapping area at 3.965 ppm. Chemical shift indicates the chemical shift of the spectral buckets used for multivariate analysis.

PCA was performed using SIMCA-P+ 12.0.1 software (UMETRICS, Umeå, Sweden) after centering and pareto-scaling of the data as previously described [23]. In addition, MLPLS-DA was performed using Matlab (version 2009a, MathWorks), and in-house written Matlab routines [24,25] in order to account for the crossover design and the possibility of pair-wise comparison of multivariate data in participants after RB and WB treatments. MLPLS-DA can be considered a multivariate extension of a paired *t*-test that generates different multivariate sub-models for between-subject and within-subject variation (intervention effect) in the data [24,25]. The advantage of this variation splitting is that each submodel can be analyzed separately without being confounded by the other source of variation. Rank product provided by MLPLS-DA analysis was then used to determine the most important NMR signals in the multilevel classification model [24,25]. The multivariate data were pareto-scaled by dividing each variable by the square root of its standard deviation. The validity of MLPLS-DA was tested by one thousand permutation to determine the *H*_0_ distribution of no-effect (P<0.05) [24,25]. The spectral variable with the highest PLS regression coefficient is assigned the lowest rank product (reported on logarithmic scale) and possesses the largest discriminatory power [24,25] (Figure 1). The absolute concentrations of the metabolites, which their corresponding NMR signals were found discriminative in MLPLS-DA, were calculated from the NMR spectra using NMR Suite 7.1 profiler (ChenomX Inc, Edmonton, Canada) and internal standard after correction for overlapping signals. Paired *t*-test was then performed on the absolute concentrations of four discriminative metabolites between RB Vs WB [26]. Paired *t*-test as a univariate approach does not take covariance between these metabolites into account. The presence of outliers was investigated using PCA-Hotelling T^2^ Ellipse (95% CI) and the normality of multivariate data was investigated using the normal probability plot of PCA model. The multivariate data were normally distributed. The values for absolute concentrations of the discriminative metabolites between RB Vs WB were log-transformed before the paired *t-*test when the distribution was skewed (Anderson-Darling test, p<0.05).

Differences in energy, nutrient, and fiber intake and in biochemical variables, between and within the test bread periods, were analyzed with paired t-tests. All results are expressed as mean ± SD. Differences were considered statistically significant at p<0.05.

## Results

Overall, the participants complied well with the study. Test bread intake exceeded the minimum number of portions recommended during both bread periods (8 vs. 4 portions/day). Total energy and fat intake did not differ between the bread periods, while protein, carbohydrate, and dietary fiber intake was higher after RB than after WB (p<0.05) (Table 1). Interestingly, at the end of both RB and WB period, total fat, SFA, MUFA, and PUFA intake was lower (p<0.05) than at the end of the run-in period.

**Table 1:** **Daily energy, macronutrient, and fiber intake by postmenopausal women during the run-in, high-fiber rye bread (RB) and refined wheat bread (WB) periods, and test bread intake**^**1**^

	**Run-in**	**RB**	**WB**
Energy (MJ/d)	7.2 ± 1.5	7.5 ± 1.5	7.3 ± 1.5
Protein (% energy)	17.4 ± 3.2	18.4 ± 2.2^a^	17.3 ± 2.4
Total fat (% energy)	31.4 ± 6.3	27.9 ± 6.9^b^	28.9 ± 6.3^b^
SFA (% energy)	13.3 ± 3.5	11.4 ± 3.5^b^	11.3 ± 3.3^b^
MUFA (% energy)	10.4 ± 2.5	9.0 ± 2.9^b^	8.5 ± 2.8^b^
PUFA (% energy)	5.0 ± 1.1	3.5 ± 1.1^b^	3.6 ± 1.4^b^
Carbohydrates (% energy)	49.5 ± 6.9	52.8 ± 6.2^a,b^	52.5 ± 7.2^b^
Total dietary fiber (g)	24.2 ± 6.9	47.2 ± 9.1^a,b^	15.4 ± 4.0^b^
Insoluble dietary fiber (g)	11.01 ± 3.2	33.4 ± 6.5 ^a,b^	6.2 ± 1.2^b^
Soluble dietary fiber (g)	5.6 ± 1.8	9.4 ± 1.9 ^a,b^	4.9 ± 1.4^b^
Cholesterol (mg)	231 ± 111	228 ± 99	198 ± 84
Minimum amount of test breads to be consumed (g)		117 ± 18	103 ± 16
Test bread intake (g)		215 ± 47	180 ± 42

^1^Values are expressed as mean ± SD; *n* = 33. Intake was calculated from 4-d food records.^a^*P*<0.05 compared with WB; ^b^*P*<0.05 compared with run-in (paired t-test).

MLPLS-DA was performed on the values of spectral data (buckets) of each subject after RB Vs WB (n=33) and was tested for the validity of the model (*H*_0_ distribution of no-effect-P<0.05). The absolute concentrations of four metabolites, which their corresponding NMR signals were found discriminative based on their rank product in MLPLS-DA (Figure 1), were calculated from the NMR spectra and further investigated by paired *t*-test (Table 2). Plasma levels of leucine and isoleucine were lower and plasma levels of betaine and N,N-dimethylglycine were higher after RB than WB. Regarding the biochemical characteristics of the postmenopausal women, total serum cholesterol and LDL cholesterol levels were higher (p<0.05) after RB than after WB (Table 3), while no significant differences were observed between serum HDL cholesterol and triglycerides after RB compared with WB. A slight but significant reduction in body weight (p<0.05) was observed within the RB group, but there was no significant difference in body weight after RB compared with WB.

**Table 2:** **Results of the NMR-based metabolomics analysis of the serum of postmenopausal women after intervention with rye and refined wheat breads**^**1**^

**Metabolite**	**NMR signal (ppm)**^**2**^	**Rank product (NMR signal)**^**3**^	**Concentration (μmol/L)**^**4**^**control vs rye**	**P-value**^**5**^
Isoleucine	0.924, 0.943, 0.965	54.9, -, -^6^	65.7 ± 10.5 Vs 61.3 ± 9.8	0.005
Leucine	0.952, 0.965	-, -^6^	128.9 ± 14.3 Vs 121 ± 13.5	0.007
Betaine	3.273	7.9	24.9 ± 1.9 Vs 26.3 ± 3.1	0.005
N,N-dimethylglycine	2.930	23.6	3.27 ± 0.58 Vs 3.6 ± 0.90	0.002^7^

Leucine and Isoleucine levels were lower and betaine and N,N-dimethylglycine levels were higher after the intake of rye bread compared with the intake of refined wheat bread (control).

^1^ n = 33.

^2^NMR signals were identified using NMR Suite 7.1 library (ChenomX Inc, Edmonton, Canada), Human Metabolome Data Base, Biological Magnetic Resonance Data Bank, spiking with an authentic standard, and confirmed with 2D NMR in the event of multiplicity.

^3^The values of rank product are reported on a logarithmic scale [24] and referee to NMR signals found discriminative using MLPLS-DA.

^4^The absolute concentration of the metabolites in serum was calculated from area under their NMR signals using NMR Suite 7.1 profiler (ChenomX Inc, Edmonton, Canada) and internal standard after correcting for overlapping signals.

^5^Paired t-test was performed on the absolute concentrations of the metabolites their NMR signals were found discriminative between two treatments.

^6^Isoleucine and leucine possess common and separate NMR signals in a small spectral region (0.924-0.965 ppm), and therefore all of their NMR signals between 0.924-0.965 ppm were tested using paired t-test (P<0.05), when an isoleucine signal at 0.924 was detected as discriminative by MLPLS-DA (RP=54.9).

^7^The p-value was calculated using log-transformed data.

**Table 3:** **Characteristics of the postmenopausal women before and after the RB and WB periods**^**1**^

	**RB**		**WB**	
	**Baseline**	**Endpoint**	**Baseline**	**Endpoint**
Body weight (kg)	69.9 ± 8.1^a^	69.5 ± 8.1	69.8 ± 8.2	69.7 ± 8.1
Serum total cholesterol (mmol/L)	6.5 ± 0.7^a^	6.8 ± 0.8^b^	6.5 ± 0.7	6.4 ± 0.7
Serum LDL-cholesterol (mmol/L)	4.3 ± 0.6 ^a^	4.5 ± 0.8	4.2 ± 0.7	4.1 ± 0.6 ^b^
Serum HDL-cholesterol (mmol/L)	1.7 ± 0.4	1.7 ± 0.4	1.7 ± 0.3	1.6 ± 0.3
Serum triglycerides (mmol/L)	1.3 ± 0.4	1.4 ± 0.5	1.3 ± 0.4	1.4 ± 0.4

^1^ Values expressed as mean ± SD; (n = 33);

^a^ Significantly different from endpoint within group (p<0.05; paired t test);

^b^ Significantly different from endpoint between groups (p<0.05; paired t test);

RB: high fiber rye bread, WB: refined wheat bread.

## Discussion

Epidemiological studies have consistently shown that WG cereals can protect against the development of chronic disease, e.g. CVD and T2D [2-10,27]. However, the underlying molecular mechanisms linking WG intake and the beneficial effects observed in epidemiological studies are not fully understood [27]. Metabolomics has proven successful in explaining some of the beneficial effects of WG products on health [15-18]. However, wider understanding of the beneficial effects of WG can be achieved by evaluating the metabolic impact of WG in different target populations. In the present study, NMR-based metabolomics was used to study the metabolic effects of intervention with rye bran-enriched rye bread (RB) in postmenopausal women with slightly elevated serum cholesterol. The overall aim was to elucidate the metabolic effects of RB in a new target group as a mechanistic approach for further understanding the beneficial effects of rye on health.

We used a quantitative NMR-based metabolomics analysis in which the concentrations of the serum metabolites were measured relative to an internal standard (TSP). This approach improves the validity of findings considering the reproducible nature of ^1^H-NMR analysis. We used different approaches to confirm the identity of metabolites. In addition, all discriminating metabolites between RB and WB reported in the present study possess well-cited NMR signals [22].

### Effects of RB on branched amino acids

In the postmenopausal women studied, plasma levels of two branched chain amino acids (BCAA) were lower after intervention with RB than with WB. This is consistent with previous findings by Lankinen et al. [17] from metabolomics analysis in the FUNGENUT study, where a reduction in isoleucine was observed in a rye bread-pasta group compared with a parallel oat and wheat bread-potato group after a 12-wk intervention. Recently, two large independent longitudinal cohort studies showed that high prospective plasma levels of BCAA are significantly associated with future diabetes [28]. Subjects in the top quartile had a five-fold higher risk of developing T2D, and plasma BCAA levels were even predictive among subjects with similar fasting insulin and glucose levels [28]. Interestingly, in the present study, the lower plasma BCAA after RB occurred without any significant differences in fasting insulin and glucose levels between the high-fiber RB and WB treatments [19]. These findings underscore the potentially positive effects of RB in alleviating the recently reported metabolic risk factors of T2D [28]. Supplementation studies with BCAA in animals and humans have indicated that circulating amino acids may directly promote insulin resistance, possibly via disruption of insulin signaling in the skeletal muscles. This may be caused by activation of the mammalian target of rapamycin, c-JUN, and insulin receptor substrate-1 signaling in the skeletal muscles [29-32].

The known genetic risk factors of T2D add slightly to the risk [33,34] and are associated with only a 5-37% increase in the relative risk of diabetes, compared with the 60-100% increase in risk that amino acids can predict [28]. This highlights the significance of final readout of genetic and environmental factors in development of T2D, which is reflected in the subject’s metabolic profile. Our findings indicate that high-fiber RB intake potentially causes a favorable shift in the metabolic profile of postmenopausal women by reducing the levels of some of circulating BCAAs known to be associated with increased incidence of T2D.

Consumption of RB, either as endosperm or WG rye bread, evokes a significantly lower postprandial insulin response than intake of refined WB, without any change in glycemia, an effect known as the ‘rye factor’ [35-39]. Repeated reduced insulin response has been associated with reduced incidence of obesity and T2D [40]. In obese and non-obese subjects, fasting concentration of BCAA correlates with obesity and serum insulin. These correlations between obesity, insulin, and BCAA may support the notion that the lower BCAA observed in the present study can be explained by reduced postprandial insulin secretion during RB, a topic which warrants further investigation [14].

Higher levels of ketone bodies, reported in our previous intervention with rye products compared with refined wheat products in prostate cancer patients [15], were not observed in the present study. This might be related to the much lower daily intake of rye products in the present study than in the previous study (120 g/d compared with 485 g/d), gender differences, and the fact that the prostate cancer patients received 20% more energy during intervention periods than baseline in order to increase their rye intake [15].

### Effect of RB on homocysteine and cholesterol metabolism

Rye bran is a rich source of betaine [41], which may explain the increased betaine levels after rye products observed in the present and previous human and animal studies [15,42,43]. Health beneficial effects of betaine have been recently reported [44]. Betaine acts as a methyl donor in the betaine-homocysteine methyl transferase reaction (BHMT-R), which converts homocysteine and betaine to methionine and N,N-dimethylglycine [45]. In the present study, we also observed an increase in plasma N,N-dimethylglycine after the RB period, which may indicate an increase in BHMT-R. Homocysteine and methionine, the other metabolites involved in this pathway, are present in low concentrations and have overlapping signals with other metabolites on the NMR spectrum, which obstructs their measurement by NMR. The increase in betaine and N,N-dimethylglycine is consistent with our recent findings in a metabolomics study of the effect of WG rye intervention on prostate cancer patients [15]. In that study, complementary targeted analysis of homocysteine in plasma showed that the increases in betaine and N,N-dimethylglycine levels after intake of rye products were accompanied by a reduction in plasma homocysteine. However, in the present study targeted analysis of homocysteine in plasma was not possible for all the subjects because non-availability of samples.

BHMT-R is catalyzed by betaine homocysteine methyl transferase (BHMT), which is down-regulated by insulin [46]. Therefore, the shift in BHMT-R after the RB period may be attributable to reduced insulin secretion, most likely due to the rye factor [35-39], and to higher availability of betaine as a reaction precursor. Elevated circulating homocysteine levels are an independent risk factor for CVD [47-50]. In addition to the favorable effects on BHMT-R, high betaine levels have been shown to increase circulating LDL cholesterol and triacylglycerol concentrations [50]. This may explain the higher levels of total and LDL cholesterol after RB observed in the present study in postmenopausal women with slightly elevated circulating cholesterol levels. WG products have high content of dietary fiber, which can potentially reduce the reabsorption of bile acids from the intestine and therefore increase bile acid biosynthesis from cholesterol, which may eventually reduce circulating cholesterol levels. However, our findings suggest that the net effect of betaine and dietary fiber in the modulation of cholesterol levels after WG intake should be considered and warrants further investigation [50].

## Conclusion

The NMR metabolomics approach in the present study revealed notable metabolic shifts related to changes in BCAA and betaine-related metabolites (single carbon metabolism) after an intervention with high-fiber RB in postmenopausal women, a population group at risk of developing T2D. These two metabolic pathways are known to be associated with the development of chronic disease. The changes in BCAA in the present study link the potentially beneficial metabolic effects of RB to metabolic pattern, which has been associated with the development of T2D in large epidemiological studies.

## Abbreviations

BHMT: Betaine homocysteine methyl transferase; BHMT-R: Betaine-homocysteine methyl transferase reaction; BCAA: Branched chain amino acids; CVD: Cardiovascular disease; MLPLS-DA: Multilevel partial least squares-discriminant analysis; PCA: Principal component analysis; RF: Rye factor; T2D: Type 2 diabetes; RB: High fiber rye bread; WG: Whole grain; WB: Refined white bread.

## Competing interests

There is no conflict of interest.

## Authors’ contribution

HM and KP designed the intervention; KJ conducted the intervention; AM designed and supervised the NMR metabolomics analyses and performed metabolomics data analyses; NA conducted the NMR-based metabolomics analyses; AM and IB-P analyzed the biochemical data and wrote the paper; HM and KH reviewed the paper; AM had primary responsibility for the final content. All authors read and approved the final manuscript.

## Authors’ information

This work was conducted within the Nordic Centre of Excellence ‘Nordic Health - Whole Grain Food’ (HELGA) project, which is funded by five Nordic research councils. The intervention was supported by Fazer Bakeries Ltd, Vaasan & Vaasan Oy, the Technology Development Center of Finland, and the analyses by the strategic fund from the Swedish University of Agricultural Sciences for promoting metabolomics based research.

## References

[B1] Fardet A New hypotheses for the health-protective mechanisms of whole-grain cereals: what is beyond fibre? Nutr Res Rev 2010 23 65 134 10.1017/S0954422410000041 20565994

[B2] De Munter JS Hu FB Spiegelman D Franz M Van Dam RM Whole grain, bran, and germ intake and risk of type 2 diabetes: a prospective cohort study and systematic review PLoS Med 2007 4 e261 10.1371/journal.pmed.0040261 17760498 PMC1952203

[B3] Murtaugh MA Jacobs DR Jacob B Steffen LM Marquart L Epidemiological support for the protection of whole grains against diabetes Proc Nutr Soc 2003 62 143 149 10.1079/PNS2002223 12740069

[B4] Jacobs DR Andersen LF Blomhoff R Whole-grain consumption is associated with a reduced risk of noncardiovascular, noncancer death attributed to inflammatory diseases in the Iowa Women’s Health Study Am J Clin Nutr 2007 85 1606 1614 17556700 10.1093/ajcn/85.6.1606

[B5] Mellen PB Walsh TF Herrington DM Whole grain intake and cardiovascular disease: a meta-analysis Nutr Metab Cardiovasc Dis 2008 18 283 290 10.1016/j.numecd.2006.12.008 17449231

[B6] Flint AJ Hu FB Glynn RJ Jensen MK Franz M Sampson L Rimm EB Whole grains and incident hypertension in men Am J Clin Nutr 2009 90 493 498 10.3945/ajcn.2009.27460 19571218 PMC2728640

[B7] Larsson SC Giovannucci E Bergkvist L Wolk A Whole grain consumption and risk of colorectal cancer: a population-based cohort of 60,000 women Br J Cancer 2005 92 1803 1807 10.1038/sj.bjc.6602543 15827552 PMC2362029

[B8] Chan JM Wang F Holly EA Whole grains and risk of pancreatic cancer in a large population-based case–control study in the San Francisco Bay Area, California Am J Epidemiol 2007 166 1174 1185 10.1093/aje/kwm194 17881383

[B9] Schatzkin A Park Y Leitzmann MF Hollenbeck AR Cross AJ Prospective study of dietary fiber, whole grain foods, and small intestinal cancer Gastroenterology 2008 135 1163 1167 10.1053/j.gastro.2008.07.015 18727930 PMC3513331

[B10] Haas P Machado MJ Anton AA Silva AS De Francisco A Effectiveness of whole grain consumption in the prevention of colorectal cancer: Meta-analysis of cohort studies Int J Food Sci Nutr 2009 60 1 13 10.1080/09637480802183380 19306224

[B11] Rosen LA Silva LO Andersson UK Holm C Ostman EM Bjorck IM Endosperm and whole grain rye breads are characterized by low post-prandial insulin response and a beneficial blood glucose profile Nutr J 2009 8 42 10.1186/1475-2891-8-42 19781071 PMC2761418

[B12] Olsen A Egeberg R Halkjaer J Christensen J Overvad K Tjonneland A Healthy aspects of the Nordic diet are related to lower total mortality J Nutr 2011 141 639 644 10.3945/jn.110.131375 21346102

[B13] Szmuilowicz ED Stuenkel CA Seely EW Influence of menopause on diabetes and diabetes risk Nat Rev Endocrinol 2009 5 553 558 10.1038/nrendo.2009.166 19687788

[B14] Adams SH Emerging perspectives on essential amino acid metabolism in obesity and the insulin-resistant state Adv Nutr 2011 2 445 456 22332087 10.3945/an.111.000737 PMC3226382

[B15] Moazzami AA Zhang JX Kamal-Eldin A Aman P Hallmans G Johansson JE Andersson SO Nuclear Magnetic Resonance-Based Metabolomics Enables Detection of the Effects of a Whole Grain Rye and Rye Bran Diet on the Metabolic Profile of Plasma in Prostate Cancer Patients J Nutr 2011 141 2126 2132 10.3945/jn.111.148239 22013201

[B16] Lankinen M Schwab U Seppanen-Laakso T Mattila I Juntunen K Mykkanen H Poutanen K Gylling H Oresic M Metabolomic analysis of plasma metabolites that may mediate effects of rye bread on satiety and weight maintenance in postmenopausal women J Nutr 2011 141 31 36 10.3945/jn.110.131656 21084654

[B17] Lankinen M Schwab U Gopalacharyulu PV Seppanen-Laakso T Yetukuri L Sysi-Aho M Kallio P Suortti T Laaksonen DE Gylling H Dietary carbohydrate modification alters serum metabolic profiles in individuals with the metabolic syndrome Nutr Metab Cardiovasc Dis 2010 20 249 257 10.1016/j.numecd.2009.04.009 19553094

[B18] Fardet A Canlet C Gottardi G Lyan B Llorach R Remesy C Mazur A Paris A Scalbert A Whole-grain and refined wheat flours show distinct metabolic profiles in rats as assessed by a 1H NMR-based metabonomic approach J Nutr 2007 137 923 929 17374655 10.1093/jn/137.4.923

[B19] Juntunen KS Laaksonen DE Poutanen KS Niskanen LK Mykkanen HM High-fiber rye bread and insulin secretion and sensitivity in healthy postmenopausal women Am J Clin Nutr 2003 77 385 391 12540398 10.1093/ajcn/77.2.385

[B20] Tiziani S Einwas AH Lodi A Ludwig C Bunce CM Viant MR Gunther UL Optimized metabolite extraction from blood serum for H-1 nuclear magnetic resonance spectroscopy Anal Biochem 2008 377 16 23 10.1016/j.ab.2008.01.037 18312846

[B21] Hwang TL Shaka AJ Water Suppression That Works - Excitation Sculpting Using Arbitrary Wave-Forms and Pulsed-Field Gradients J Magn Reson Series A 1995 112 275 279 10.1006/jmra.1995.1047

[B22] Psychogios N Hau DD Peng J Guo AC Mandal R Bouatra S Sinelnikov I Krishnamurthy R Eisner R Gautam B The human serum metabolome PLoS One 2011 6 e16957 10.1371/journal.pone.0016957 21359215 PMC3040193

[B23] Moazzami AA Andersson R Kamal-Eldin A Changes in the metabolic profile of rat liver after alpha-tocopherol deficiency as revealed by metabolomics analysis NMR Biomed 2011 24 499 505 10.1002/nbm.1615 21674651

[B24] van Velzen EJ Westerhuis JA van Duynhoven JP van Dorsten FA Hoefsloot HC Jacobs DM Smit S Draijer R Kroner CI Smilde AK Multilevel data analysis of a crossover designed human nutritional intervention study J Proteome Res 2008 7 4483 4491 10.1021/pr800145j 18754629

[B25] Westerhuis JA Van Velzen EJ Hoefsloot HC Smilde AK Multivariate paired data analysis: multilevel PLSDA versus OPLSDA Metabolomics 2010 6 119 128 10.1007/s11306-009-0185-z 20339442 PMC2834771

[B26] Yde CC Westerhuis JA Bertram HC Bach Knudsen KE Application of NMR-based metabonomics suggests a relationship between betaine absorption and elevated creatine plasma concentrations in catheterised sows Br J Nutr 2012 107 1603 1615 10.1017/S0007114511004909 22673149

[B27] Slavin JL Jacobs D Marquart L Wiemer K The role of whole grains in disease prevention J Am Diet Assoc 2001 101 780 785 10.1016/S0002-8223(01)00194-8 11478475

[B28] Wang TJ Larson MG Vasan RS Cheng S Rhee EP McCabe E Lewis GD Fox CS Jacques PF Fernandez C Metabolite profiles and the risk of developing diabetes Nat Med 2011 17 448 453 10.1038/nm.2307 21423183 PMC3126616

[B29] Felig P Marliss E Cahill GF Plasma amino acid levels and insulin secretion in obesity N Engl J Med 1969 281 811 816 10.1056/NEJM196910092811503 5809519

[B30] Patti ME Brambilla E Luzi L Landaker EJ Kahn CR Bidirectional modulation of insulin action by amino acids J Clin Invest 1998 101 1519 1529 10.1172/JCI1326 9525995 PMC508730

[B31] Newgard CB An J Bain JR Muehlbauer MJ Stevens RD Lien LF Haqq AM Shah SH Arlotto M Slentz CA A branched-chain amino acid-related metabolic signature that differentiates obese and lean humans and contributes to insulin resistance Cell Metab 2009 9 311 326 10.1016/j.cmet.2009.02.002 19356713 PMC3640280

[B32] Krebs M Krssak M Bernroider E Anderwald C Brehm A Meyerspeer M Nowotny P Roth E Waldhausl W Roden M Mechanism of amino acid-induced skeletal muscle insulin resistance in humans Diabetes 2002 51 599 605 10.2337/diabetes.51.3.599 11872656

[B33] Meigs JB Shrader P Sullivan LM McAteer JB Fox CS Dupuis J Manning AK Florez JC Wilson PW D’Agostino RB Cupples LA Genotype score in addition to common risk factors for prediction of type 2 diabetes N Engl J Med 2008 359 2208 2219 10.1056/NEJMoa0804742 19020323 PMC2746946

[B34] Lyssenko V Jonsson A Almgren P Pulizzi N Isomaa B Tuomi T Berglund G Altshuler D Nilsson P Groop L Clinical risk factors, DNA variants, and the development of type 2 diabetes N Engl J Med 2008 359 2220 2232 10.1056/NEJMoa0801869 19020324

[B35] Leinonen K Liukkonen K Poutanen K Uusitupa M Mykkanen H Rye bread decreases postprandial insulin response but does not alter glucose response in healthy Finnish subjects Eur J Clin Nutr 1999 53 262 267 10.1038/sj.ejcn.1600716 10334650

[B36] Juntunen KS Laaksonen DE Autio K Niskanen LK Holst JJ Savolainen KE Liukkonen KH Poutanen KS Mykkanen HM Structural differences between rye and wheat breads but not total fiber content may explain the lower postprandial insulin response to rye bread Am J Clin Nutr 2003 78 957 964 14594782 10.1093/ajcn/78.5.957

[B37] Kallio P Kolehmainen M Laaksonen DE Pulkkinen L Atalay M Mykkanen H Uusitupa M Poutanen K Niskanen L Inflammation markers are modulated by responses to diets differing in postprandial insulin responses in individuals with the metabolic syndrome Am J Clin Nutr 2008 87 1497 1503 18469276 10.1093/ajcn/87.5.1497

[B38] Juntunen KS Niskanen LK Liukkonen KH Poutanen KS Holst JJ Mykkanen HM Postprandial glucose, insulin, and incretin responses to grain products in healthy subjects Am J Clin Nutr 2002 75 254 262 11815315 10.1093/ajcn/75.2.254

[B39] Bondia-Pons I Nordlund E Mattila I Katina K Aura AM Kolehmainen M Oresic M Mykkanen H Poutanen K Postprandial differences in the plasma metabolome of healthy Finnish subjects after intake of a sourdough fermented endosperm rye bread versus white wheat bread Nutr J 2011 10 116 10.1186/1475-2891-10-116 22011443 PMC3214176

[B40] Ludwig DS The glycemic index: physiological mechanisms relating to obesity, diabetes, and cardiovascular disease JAMA 2002 287 2414 2423 10.1001/jama.287.18.2414 11988062

[B41] Bruce SJ Guy PA Rezzi S Ross AB Quantitative measurement of betaine and free choline in plasma, cereals and cereal products by isotope dilution LC-MS/MS J Agric Food Chem 2010 58 2055 2061 10.1021/jf903930k 20102202

[B42] Bertram HC Bach Knudsen KE Serena A Malmendal A Nielsen NC Frette XC Andersen HJ NMR-based metabonomic studies reveal changes in the biochemical profile of plasma and urine from pigs fed high-fibre rye bread Br J Nutr 2006 95 955 962 10.1079/BJN20061761 16611386

[B43] Bertram HC Malmendal A Nielsen NC Straadt IK Larsen T Knudsen KE Laerke HN NMR-based metabonomics reveals that plasma betaine increases upon intake of high-fiber rye buns in hypercholesterolemic pigs Mol Nutr Food Res 2009 53 1055 1062 10.1002/mnfr.200800344 19603403

[B44] Wang Z Klipfell E Bennett BJ Koeth R Levison BS Dugar B Feldstein AE Britt EB Fu X Chung YM Gut flora metabolism of phosphatidylcholine promotes cardiovascular disease Nature 2011 472 57 63 10.1038/nature09922 21475195 PMC3086762

[B45] Delgado-Reyes CV Garrow TA High sodium chloride intake decreases betaine-homocysteine S-methyltransferase expression in guinea pig liver and kidney Am J Physiol Regul Integr Comp Physiol 2005 288 R182 R187 15331385 10.1152/ajpregu.00406.2004

[B46] Ratnam S Wijekoon EP Hall B Garrow TA Brosnan ME Brosnan JT Effects of diabetes and insulin on betaine-homocysteine S-methyltransferase expression in rat liver Am J Physiol Endocrinol Metab 2006 290 E933 E939 10.1152/ajpendo.00498.2005 16352668

[B47] Clarke R Daly L Robinson K Naughten E Cahalane S Fowler B Graham I Hyperhomocysteinemia: an independent risk factor for vascular disease N Engl J Med 1991 324 1149 1155 10.1056/NEJM199104253241701 2011158

[B48] Kang SS Wong PW Malinow MR Hyperhomocyst(e)inemia as a risk factor for occlusive vascular disease Annu Rev Nutr 1992 12 279 298 10.1146/annurev.nu.12.070192.001431 1503807

[B49] Vizzardi E Bonadei I Zanini G Frattini S Fiorina C Raddino R Dei Cas L Homocysteine and heart failure: an overview Recent Pat Cardiovasc Drug Discov 2009 4 15 21 10.2174/157489009787259991 19149701

[B50] Olthof MR Van Vliet T Verhoef P Zock PL Katan MB Effect of homocysteine-lowering nutrients on blood lipids: results from four randomised, placebo-controlled studies in healthy humans PLoS Med 2005 2 e135 10.1371/journal.pmed.0020135 15916468 PMC1140947

